# Photophysical and Electro-Optical Properties of Copolymers Bearing Blue and Red Chromophores for Single-Layer White OLEDs

**DOI:** 10.3390/nano11102629

**Published:** 2021-10-06

**Authors:** Despoina Tselekidou, Kyparisis Papadopoulos, Vasileios Kyriazopoulos, Konstantinos C. Andrikopoulos, Aikaterini K. Andreopoulou, Joannis K. Kallitsis, Argiris Laskarakis, Stergios Logothetidis, Maria Gioti

**Affiliations:** 1 Nanotechnology Lab LTFN, Department of Physics, Aristotle University of Thessaloniki, GR-54124 Thessaloniki, Greece; detselek@physics.auth.gr (D.T.); kypapado@physics.auth.gr (K.P.); vkyriazo@physics.auth.gr (V.K.); alask@physics.auth.gr (A.L.); logot@auth.gr (S.L.); 2 Organic Electronic Technologies P.C. (OET), Antoni Tritsi 21B, GR-57001 Thessaloniki, Greece; 3 Department of Chemistry, University of Patras, Caratheodory 1, University Campus, GR-26504 Patras, Greece; k.andrikopoulos@upnet.gr (K.C.A.); andreopo@upatras.gr (A.K.A.); kallitsi@upatras.gr (J.K.K.)

**Keywords:** carbazole, benzothiadiazole, energy transfer, WOLED, spin coating, copolymer, ellipsometry, photoluminescence, electroluminescence, synthesis

## Abstract

In this study, novel copolymers consisting of blue and red chromophores are presented to induce emission tuning, enabling the definition of white light emission in a single polymeric layer. These aromatic polyether sulfones exhibit high molecular weights, excellent solubility and processability via solution deposition techniques. In addition, by carefully controlling the molar ratios of chromophores composition, the energy transfer mechanism, from blue to red chromophores, takes place enabling us to define properly the emission covering the entire range of the visible spectrum. The optical and photophysical properties of the monomers and copolymers were thoroughly investigated via NIR-Vis-far UV Spectroscopic Ellipsometry (SE), Absorbance and Photoluminescence (PL). These copolymers are used as an emissive layer and applied in solution-processed WOLED devices. The fabricated WOLED devices have been subsequently studied and characterized in terms of their electroluminescence properties. Finally, the WOLED devices possess high color stability and demonstrate CIE Coordinates (0.33, 0.38), which approach closely the pure white light CIE coordinates.

## 1. Introduction

In recent years, tremendous advances in the field of Polymer Light-Emitting Diodes (PLEDs) have been achieved mainly through the synthesis of novel emitting materials and the development of improved device architectures [1,2,3,4,5]. In particular, scientific interest has largely increased in White Organic Light-Emitting Diodes (WOLEDs) processed from wet-based techniques, which have strong potential for use in future solid-state light sources and flat panel displays [1,2,3,4,5,6,7,8,9]. The wet-based fabrication process of such devices is compatible with automated roll-to-roll coating, which can allow upscaling to large scale industrial production of these devices [1,4,7].

A variety of approaches based on semiconductor polymers, as the active layer of wet-processed OLEDs, have been proposed for the realization of white light emission [5]. Most of the WOLED devices reported so far have relied on the use of combinations of several organic components in blended systems or heterolayer architectures that emit different colors to fully span the entire visible spectrum [5]. However, the preparation of such blended or heterolayer devices is more complicated; in the case of blended systems, there is a probability of phase separation within the films, while regarding the heterolayer structure, the intermixing of the different layers may harm the previously deposited layer [5,10,11].

Compared to the multi-emitting-component WOLEDs, a promising alternative strategy has been proposed with the fabrication of a single-layer WOLED, based on the inclusion of copolymers that bear different emitting chromophores [10,11,12]. This approach has many advantages, such as a simple and cost-effective fabrication process, and utilization in spin-coating and printing methods over a large-scale range due to the easy processability of copolymers in common solvents [11,12].

In addition, an active area of research has been carried out with the objective of design and synthesis of new copolymers, which combine the excellent electrical and optical properties of inorganic semiconductors with the advantages of polymeric materials, such as low density, easy processability, and synthetic tunability [4,13,14]. Many conjugated polymers composed of complementary (blue and yellow or orange) or primary colours (red, green, and blue), have been synthesized to achieve the emission tuning of different emitting chromophores, enabling the definition of white light emission in a polymeric layer [5,11].

However, the mechanisms of white emission by using copolymers that simultaneously bear three chromophores of different color bring about more difficulty in accurately understanding and controlling the energy transfer from host-like to guest-like units through the chromophore’s concentrations [11,12]. Two-color white electroluminescent polymers are easier to develop and may achieve higher efficiencies than the three-color white electroluminescent polymers, due to fewer trap states along the polymer backbone and more simple energy transfer processes in-between the differently colored chromophores. Therefore, the copolymers bearing two-color chromophores are promising candidates as emissive layers in WOLED devices in terms of improving the control over the Commission Internationale de l’Eclairage (CIE) x, y color coordinates and finally achieving white light emission.

In a host–guest system, by controlling the molar ratios of the chromophores, one may obtain different emission colors and as a result to cover the visible spectrum. In analogy, the emission color of copolymers can be tuned in the entire visible range through two competing mechanisms which are the energy transfer and the charge trapping. More specifically, in Fluorescence Energy Transfer (FET) process, an excited fluorophore (host) transfers its energy to an adjacent molecule (guest) by dipole–dipole interaction. Guest–host systems are also attractive materials for investigating energy-migration processes [10,11,12,13,14,15,16,17,18]. There are two types of FET processes, which are dependent on the copolymer chain architecture, host/guest strength and composition. The first one is intra-chain FET, i.e., hopping of electronic excitations along a polymeric chain and the other is inter-chain FET between copolymer chains [11]. To fully exploit the advantages of the copolymerization strategy, it is necessary to understand this interplay between host-guest copolymer chain structure and the optical and electronic properties, as well. Furthermore, all these mechanisms result in the partial or complete quenching of the host emission. Therefore, the need for tuning the chemical structure of emitting copolymers as well as to design simple-structured white OLEDs remains an open issue and triggers the advanced research for establishing the use of novel and stable white light emitting materials.

In the present study, the novel copolymers consisting of blue and red chromophores are presented and applied as emissive layers in WOLEDs. The aromatic polyethers synthesized contain 2,6- diphenyl pyridine, along with two chromophores, namely, 2,7- distyryl carbazole (Cz) and 4,7- distyryl benzothiadiazole (BTZ) as blue and red emitting chromophores, respectively. The fruitful combination of the chromophores along with the appreciable high molecular weights of the copolymers, without sacrificing their solubility, and their processability in the form of solution inks, allows for optimum deposition and study of the photoactive layers. The ease of the monomers’ preparation [10,19,20], the versatility and tolerance of the polymerization procedure [21,22] and the efficient and straightforward purification of the final semiconductive polyethers demonstrate the economic viability of the herein presented materials compared to analogous commercial semiconducting fully conjugated polymers. In order to identify their emission characteristics, we have compared white emitting copolymers with blue emitting polymers bearing either only carbazole or both carbazole and pyridine moieties in the polymeric chain. We focus on the complete photophysical and optical characterization of the emissive films to clarify the energy transfer mechanism between the two chromophores. Furthermore, with precise control over the molecular ratio distribution of the emitting chromophores, we achieved emission in the entire visible region. The possibilities for the application of the proposed WOLED technology are investigated and discussed. The electrical characteristics of the produced WOLED devices are reported, as well. Finally, the fabricated devices exhibit superior color stability with CIE Coordinates (0.33, 0.38).

## 2. Materials and Methods

### 2.1. General Synthetic Procedure

Monomers 2,6-dihydroxyphenylpyridine (Py) [19], 2,7-diacetoxystyryl-9-(2-ethylhexyl)-9H-carbazole (Cz) and 4,7-diacetoxystyryl-2,1,3-benzothiadiazole (BTZ) [10,20] were synthesized according to the literature procedures. Dimethylacetamide (DMA) and toluene were purchased from Honeywell and were used as received. K_2_CO_3_ was purchased from Penta (Radiová 1122/110200 Prague 10, Czech Republic) and was used without further purification. Difluorophenylsulfone was purchased from Alfa Aesar (Thermo Fisher (Kandel) GmbH Postfach, Karlsruhe, Germany).

General polymerization procedure: To an appropriate round-bottom flask, equipped with a magnetic stirrer, after it was flamed and purged with argon, the appropriate ratios of Cz (*x* mol), BTZ (*y* mol) and Py (*z* mol) were added. Difluorophenylsulfone (*x* + *y* + *z* mol) was also added, along with K_2_CO_3_ (2 (*x* + *y* + *z*) mol). DMA and toluene were then added, the mixture was thoroughly degassed and filled with Ar and finally the flask was fitted with a Dean–Stark apparatus. The polymerization mixture was stirred at 150 °C for 4 h. Then, the temperature was increased to 165 °C and an azeotropic mixture of toluene- water was removed. The reaction was left stirring for a further 4 h before it was precipitated in a 10-fold excess of ethanol. The precipitated copolymer was washed with ethanol and was left stirring overnight in ethyl acetate. The copolymer was then filtered, washed thoroughly with warm water and finally with ethanol. The obtained solid was then dried under vacuum at 40 °C to afford the final copolymer.

WCop1 (Py50-Cz45-BTZ5). Cz (59.12 mg, 0.0986 mmol), BTZ (5.00 mg, 0.0110 mmol), Py (28.85 mg, 0.1096 mmol), difluorophenylsulfone (55.74 mg, 0.2196 mmol), K_2_CO_3_ (37.87 mg, 0.2740 mmol), DMA 2 mL and toluene 1 mL were used in the polymerization to afford 150.00 mg. ^1^H NMR (600 MHz, CDC l_3_, *δ* ppm): 8.21–8.13 (br, 4H), 8.05–7.99 (br, 2H), 7.97–7.79 (br, 13H), 7.70–7.64 (br, 2H), 7.61–7.55 (br, 4H), 7.47–7.40 (br, 6H), 7.29–7.01 (4 br, 36H), 4.20 (br, 2H), 2.12 (br, 1H), 1.51–1.28 (2 br, 8H), 1.01–0.81 (2 br, 6H).

WCop2 (Py50Cz49.5-BTZ0.5). Cz (600 mg, 1.0004 mmol), BTZ (4.61 mg, 0.0101 mmol), Py (266.05 mg, 1.0100 mmol), difluorophenylsulfone (513.84 mg, 2.0200 mmol), K_2_CO_3_ (614.49 mg, 4.45 mmol), DMA 14 mL and toluene 4 mL were used in the polymerization to afford 1.01 g. ^1^H NMR (600 MHz, CDC l_3_, *δ* ppm): 8.24–8.12 (br, 4H), 8.08–8.00 (br, 2H), 7.98–7.82 (br, 13H), 7.76–7.65 (br, 2H), 7.65–7.54 (br, 4H), 7.52–7.39 (br, 6H), 7.31–7.01 (4 br, 36H), 4.21 (br, 2H), 1.83 (br, 1H), 1.73–1.17 (2 br, 8H), 1.01–0.84 (2 br, 6H).

CzHom (Cz100). Cz (3.00 g, 0.0050 mmol), difluorophenylsulfone (1.27 g, 0.0050 mmol), K_2_CO_3_ (1.04 g, 0.0075 mmol), DMA 12 mL and toluene 3 mL were used in the polymerization to afford 3.50 g. ^1^H NMR (600 MHz, DMF-d_7_, *δ* ppm): 8.2–8.15 (br, 2H), 8.14–8.06 (br, 4H), 7.93–7.87 (br, 2H), 7.84–7.73 (br, 4H), 7.62–7.47 (br, 6H), 7.42–7.49 (br, 8H), 4,44 (br, 2H), 2.22 (br, 1H), 1.52–1.19 (m, 8H), 0.97 (t, J = 7.16 Hz, 3H), 0.84 (t, J = 6.80 Hz, 3H).

CzCop (Cz0.5-Py0.5). Cz (300.00 mg, 0.5002 mmol), Py (131.70 mg, 0.5002 mmol), difluorophenylsulfone (254.38 mg, 1.0000 mmol), K_2_CO_3_ (207.39 mg, 1.5000 mmol), DMA 10 mL and toluene 2 mL were used in the polymerization to afford 600 mg. ^1^H NMR (600 MHz, CDC l_3_, *δ* ppm): 8.23–8.13 (br, 4H), 8.04 (s, 2H), 7.95–7.85 (br, 9H), 7.70 (d, J = 6.40 Hz, 2H), 7.60 (br, 2H), 7.49–7.42 (br, 4H), 7.27–7.02 (4 br, 22H), 4.28–4.14 (br, 2H), 2.18–2.12 (br, 1H), 1.54–1.29 (br, 4H), 1.00–0.81 (br, 10H).

### 2.2. Ink Formulation

For the Hole Transport Layer (HTL) a solution of poly-3,4-ethylene dioxythiophene: polystyrene sulfonate (PEDOT: PSS, received by Heraus, Germany) Clevios P VP AI 4083 mixed with ethanol in the ratio of 2:1 was prepared. The copolymers, of different molecular weights, were dissolved in N, N-Dimethylformamide DMF with a resulting concentration of 1 wt%.

### 2.3. OLED Fabrication

**Figure 1:**
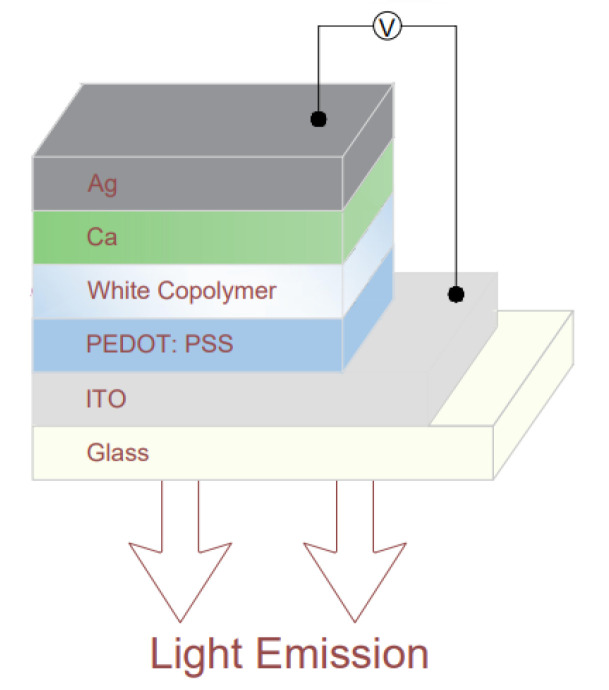
The architecture of the fabricated OLED devices.

The structure of the fabricated OLED devices is illustrated in Figure 1. Firstly, pre-patterned Indium-Tin Oxide-coated glass substrates (received by Ossila Ltd. Sheffield, UK) were extensively cleaned by sequential ultra-sonication baths in DI water with Hellmanex detergent (received by Ossila Ltd. Sheffield, UK), DI water, acetone, and ethanol for 10 min, followed by nitrogen drying. The substrates were also treated with oxygen plasma at 40 W for 3 min. Then, the PEDOT: PSS layer, which was used as the Hole Transport Layer (HTL), was deposited by spin coating method onto the glass/ITO substrate and followed by annealing at 120 °C for 5 min. The emitting layers (EML) were spun at the same speed onto the PEDOT: PSS layer. All the spin coating processes were carried out under ambient conditions. Finally, a bilayer of Ca with a thickness of 10 nm and Ag with a thickness of 100 nm, which was used as a cathode electrode bilayer, was deposited using the appropriate shadow masks by Vacuum Thermal Evaporation (VTE).

### 2.4. Thin Film and Device Characterization

The optical and electronic properties of the copolymers have been examined by Spectroscopic Ellipsometry (SE). By applying the appropriate modelling and fitting procedures we have extracted significant information about the dielectric function ε (E), the refractive index, the absorption coefficient and the thickness of the studied thin films with nanometer scale precision. The SE measurements were conducted using a UVISEL phase modulated ellipsometer (HORIBA Europe Research Center—Palaiseau, France) from the near IR to far UV spectral region 1.5–6.5 eV with a step of 20 meV at an angle of incidence of 70°. The SE experimental data were fitted to model-generated data using the Levenberg–Marquardt algorithm taking into consideration all the fitting parameters of the applied model.

Thin films of reference samples of CzHom, CzCop and BTZ, grown on glass substrates, were studied using spectroscopic specular absorbance measurements at near-normal incidence in the spectral range of 330–790 nm using a combined deuterium and halogen light source, a co-axial fiber optic reflection probe, a high line density grating and a CCD detector (by Avantes, Oude Apeldoornseweg 28, 7333 NS Apeldoorn, Netherlands).

Finally, the photoluminescence (PL) characteristics of the active layers were measured using the Hamamatsu Absolute PL Quantum Yield measurement system (C9920-02) (Joko-cho, Higashi-ku, Hamamatsu City, 431-3196, Japan), while the electroluminescence (EL) characteristics of the final OLED devices were acquired by the Hamamatsu External Quantum Efficiency system (C9920-12) (Joko-cho, Higashi-ku, Hamamatsu City, 431-3196, Japan), which measures the brightness and light distribution of the devices, as well as the current density and luminance versus voltage.

## 3. Results and Discussion

### 3.1. Copolymer Synthesis

An efficient strategy to control the solution processability and the morphology of semiconducting systems is the incorporation of spacers as conjugation-break units between the π-conjugated segments. This approach has been thoroughly investigated in the past by our group, showing excellent results over the preparation of conjugated polymers carrying different chromophores interrupted by aliphatic flexible spacers of high molecular weights, excellent film forming ability and solubility in organic solvents, control and fine-tuning of the emission light and increased thermal and mechanical stability [4,9,18,20].

**Scheme 1:**
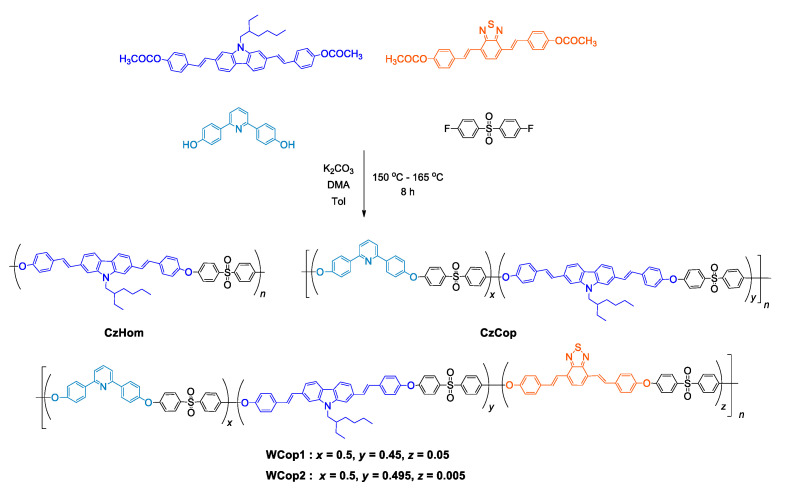
Synthetic route for the light emitting copolymers.

Herein, to obtain white light emitting polymers aromatic polyetherification condensation conditions were employed as shown in Scheme 1. The high temperature polyetherifications were performed in a high boiling point non-protic solvent, namely dimethylacetamide (DMA) using potassium carbonate (K_2_CO_3_) as the base. To this polymerization medium, toluene was also added to create an azeotropic mixture with the water formed during the polymerization and to aid the kinetics of the reaction. Aromatic polyethers are attractive photoactive materials as they can maintain control of the conjugation length of the chromophores, can achieve high molecular weights and excellent solubilities and exhibit excellent thermal and electrical stability, as has been demonstrated before. [21,22]. 2,6-Diphenyl-pyridine (Py) was chosen as a comonomer in these polymerizations acting as a solubilizing unit and also as a “host-like” element across the polymer backbone that separates and “dilutes” the emissive chromophores. We used 2,7-Distyryl carbazole (Cz) and 4,7-distyryl benzothiadiazole (BTZ) as the blue and red chromophores, respectively. Both the Py and Cz moieties impart the copolymers with high hole mobility due to their nitrogen heteroatoms. Furthermore, difluorodiphenyl sulfone was used as a comonomer in all cases serving a dual purpose and imparting two different properties. It can produce high molecular weight polymers of excellent solubility, thermal and oxidative stability [21,22] while the electron withdrawing nature of the sulfone core entails enhanced electron mobilities [23].

The ratios of the comonomers for all polymers are presented in Table 1. All copolymers exhibited appreciable to excellent solubilities in various organic solvents ranging from CHCl_3_ to NMP, depending on the comonomers’ ratio and the molecular weight of the copolymer. On the contrary, the carbazole based homopolymer CzHom had limited solubility being soluble only in DMF and NMP (up to 5% *w/v*) but scarcely soluble in DMSO (0.5% *w/v*) and 1,1,2,2-TCE (1% *w/v*). In CHCl_3_, the CzHom was practically insoluble. However, this solubility problem was fully overcome via the incorporation of the 2,6-diphenyl-pyridine unit that afforded highly soluble copolymers in common organic solvents such as CHCl_3_, THF, DMF, NMP, 1,1,2,2-TCE and DMSO. Therefore, all copolymers were characterized in terms of their molecular weight by size exclusion chromatography. As explained above, the insolubility of CzHom in CHCl_3_ restrained its molecular weight evaluation via Gel Permeation Chromatography (GPC). Only a small fraction of lower molecular weights could be solubilized in CHCl_3_ which was the eluting phase in our GPC measurements. On the other hand, CzCop, WCop1 and WCop2 provided high molecular weight polymers that were readily soluble in CHCl_3_. The results are presented in Table 2.

### 3.2. Spectroscopic Ellipsometry

**Figure 2:**
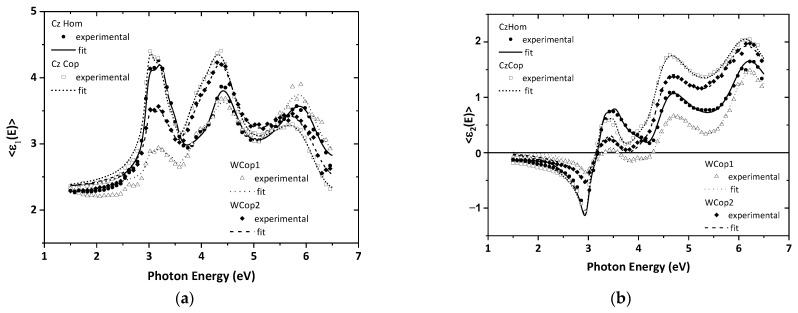
The experimental (symbols) (**a**) 〈ε_1_ (*E*)〉 and (**b**) 〈ε_2_ (*E*)〉 spectra and the corresponding fitted ones (lines) of the studied films.

SE is a non-destructive and surface sensitive technique that measures the dielectric function (ε (*E*) = ε_1_ (*E*) + iε_2_ (*E*)) and determines the optical constants of the material either in the form of a bulk sample or a thin film. In the case of a thin film in a single or multilayer structure, the measured quantity by SE is the pseudodielectric function 〈ε (*E*)〉, which also accounts the effect of the films’ thickness. The 〈ε (*E*)〉 spectra of the spin-coated films were modelled with the use of a theoretical model that consists of the layer sequence glass/ITO/PEDOT: PSS/Emitting polymer/air. For completeness, we present the study of the optical properties of CzHom and CzCop of the carbazole (Cz) compound, which is the host blue chromophore of the Cz-BTZ copolymers. Figure 2 illustrates the experimentally measured real 〈ε_1_ (*E*)〉 (a) and imaginary 〈ε_2_ (*E*)〉 (b) spectra (symbols) and the fitted ones (lines) derived by the applied fitting procedure of the spin casted films and more specifically of the film of the homopolymer carbazole derivative (CzHom), the film of the carbazole-diphenylpyridine copolymer (CzCop) and of the carbazole-benzothiadiazole- diphenylpyridine copolymers WCop1 and WCop2.

For the determination of the dielectric response of the films, we have used the Tauc–Lorentz (TL) dispersion oscillator model. In the TL model, the imaginary part *ε*_2_ (*E*) of the dielectric function is calculated by multiplying the Tauc joint density of states by the *ε*_2_ (*E*) obtained from the Lorentz oscillator model and is given by the following expressions [24]:

(1) $$\{\begin{array}{cc} \varepsilon_{2}\left(E\right)=\frac{1}{E} & \times \frac{AE_{0}\Gamma {\left(E-E_{g}\right)}^{2}}{{\left(E^{2}-E_{0}^{2}\right)}^{2}+\Gamma^{2}E^{2}},\;\;\;E>E_{g}\\ & \varepsilon_{2}\left(E\right)=0,\;\;\;E\leq E_{g} \end{array}$$

The real part $\varepsilon_{1}\left(E\right)$ is obtained by the Kramer–Kronig integration as shown below [24,25]:

(2) $$\varepsilon_{1}\left(E\right)=\varepsilon_{\infty }+\frac{2}{\pi }P\overset{\infty }{\underset{E_{g}}{\int }}\frac{\xi \varepsilon_{2}\left(\xi \right)}{\xi^{2}-E^{2}}d\xi$$

where ε∞ is the pure real part of the dielectric constant and accounts for the existence of electronic transitions that occurred at higher energies, which are not considered in the ε_2_ (*E*). The fundamental band gap *E_g_* is correlated to the energy difference between HOMO-LUMO states. The amplitude *A*, the Lorentz resonant energy *E*_0_ and the broadening term *Γ* of the oscillator are referred to the characteristics of the higher order electronic transitions. For the modelling procedure, we have deployed 6-TL oscillators to explicitly parameterize all the electronic transitions, that take place in the polymer films. In addition, the films’ thicknesses are also calculated by the fitting analysis and are shown in Table 3.

The complex refractive index of the films:

(3) $$\tilde{n}\left(E\right)=n+i\kappa$$

is then derived by the calculated dielectric function [25]:

(4) $$n=\sqrt{\frac{1}{2}\left({\left(\varepsilon_{1}^{2}+\varepsilon_{2}^{2}\right)}^{1/2}+\varepsilon_{1}\right)}$$

(5) $$\kappa =\sqrt{\frac{1}{2}\left({\left(\varepsilon_{1}^{2}+\varepsilon_{2}^{2}\right)}^{1/2}-\varepsilon_{1}\right)}$$

and the absorption coefficient is mathematically expressed as follow [25]:

(6) $$\alpha =\frac{4\pi \kappa }{\lambda }$$

**Figure 3:**
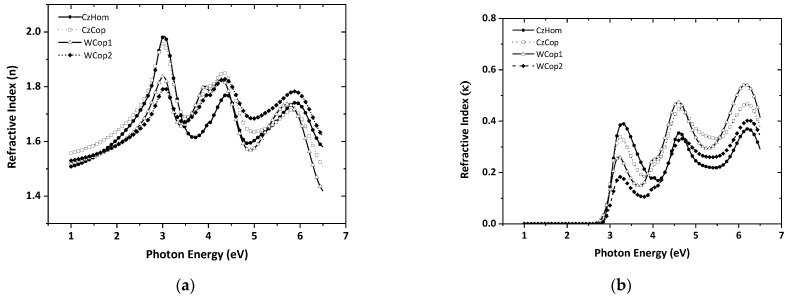
The (**a**) real n(E) and (**b**) imaginary κ(E) part, of the refractive index$\;\tilde{n}\left(E\right)$ of the studied films calculated using the best-fit parameters derived by SE analysis procedure.

Figure 3 depicts the real *n* (*E*) and the imaginary *κ* (*E*) part of the refractive index of the CzHom and CzCop as well as the two studied white emitting copolymers, using the results from the analysis procedure, described above. In terms of the characteristic electronic absorptions, which are described by the six TL oscillators, the electronic transitions in the Cz group dominate the spectra. Of note, the overall content of blue chromophores over the copolymer composition is high enough and elevates from 90 to even 99% molar ratio. It is therefore presumed that the electronic transitions of the red chromophore do not contribute effectively to the spectra, with well-defined features. For the nanostructured amorphous polymeric films, the band gap, which is derived by the TL dispersion equation $E_{g}^{TL}$, also accounting for the apparent absorption originated by the disordering and the localized defect states. For this reason, we proceeded to precise determination of the energy band gap of the produced films by Tauc plots [26], namely $E_{g}^{Tauc}$ and all the relative results are presented in Table 3. The major contribution of the blue chromophores can also be verified from the $E_{g}^{Tauc}$ value, which was calculated to be equal to 2.9–3.0 eV in all studied films.

**Figure 4:**
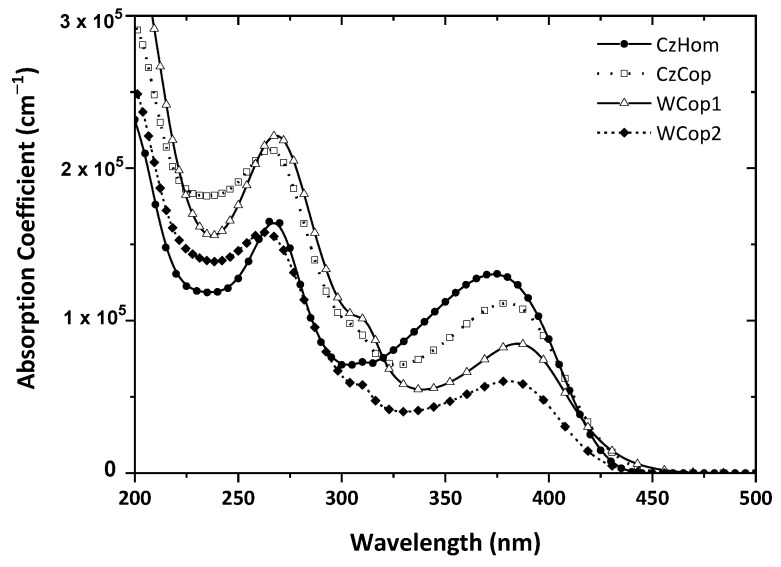
The absorption coefficient of the studied films calculated using the best-fit parameters derived by SE analysis procedure.

The absorption coefficients of CzHom, CzCop and WCop1,2 were calculated from the SE results and displayed in Figure 4. The similarities of the absorption coefficients of the Cz-based films are obvious in terms of the wavelengths where the characteristic features appear. The differences are observed in the strength of the absorption peaks as well as in the absorption edge. Indeed, for WCop1, a noticeable absorption tail was observed, which may be related to disorder-induced nanostructure and the formation of localized defect states in this film. Additionally, this is also in agreement with the calculated values of the optical band gaps $E_{g}^{TL}\;$and $E_{g}^{Tauc}$. Particularly, the differences between the$\;E_{g}^{TL}$ and$\;E_{g}^{Tauc}$ values could provide information concerning the influence of BTZ content in the WCop films’ structure. We can distinguish that the presence of BTZ units affects the value of the optical band gap $E_{g}^{TL}$, which is decreasing with the increase of the BTZ content due to the possible formation of near-band gap states [27].

### 3.3. Absorption and Photoluminescence

**Figure 5:**
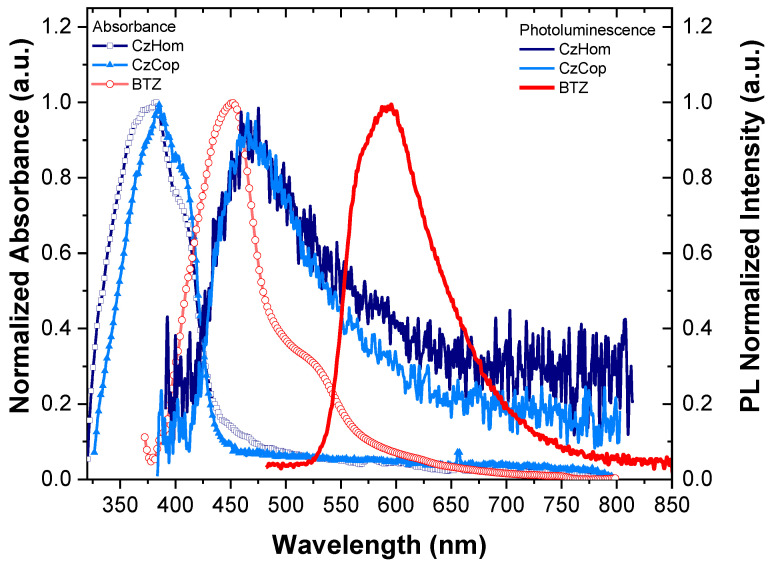
Absorption and PL spectra of CzHom, CzCop and BTZ monomer.

Figure 5 illustrates the UV-Vis Absorption and PL spectra of CzHom, CzCop and BTZ units comprising the solid thin-films. Both CzHom and CzCop exhibited maximum absorption at λ^max^~380 nm, whereas BTZ at λ^max^~450 nm. The PL emission of both CzHom and CzCop polymers was located at λ^max^~470 nm and for BTZ at λ^max^~590 nm. The comparison of UV-Vis Absorption and PL emission characteristics of the chromophores yields insights on the effectiveness of the energy transfer from blue to red chromophore, towards the final achievement of white light emission of the white copolymers. Indeed, the obtained overlap between the absorption spectra of BTZ and the emission spectra of Cz indicates a possible balanced Förster energy transfer from the Cz to BTZ segments along with the copolymer structure [28,29,30,31]. This means that the Cz unit in the electronic excited state can transfer part of its energy directly to BTZ in the electronic ground state, as it is demonstrated by the close matching of BTZ absorption spectrum over the Cz emission spectrum, at the region between 400 and 500 nm [11,12,31,32,33]. The energy transfer mechanism between donor and acceptor components is a promising approach to obtain stable color mixing in a single layer to achieve white light emission [12,31,32].

**Figure 6:**
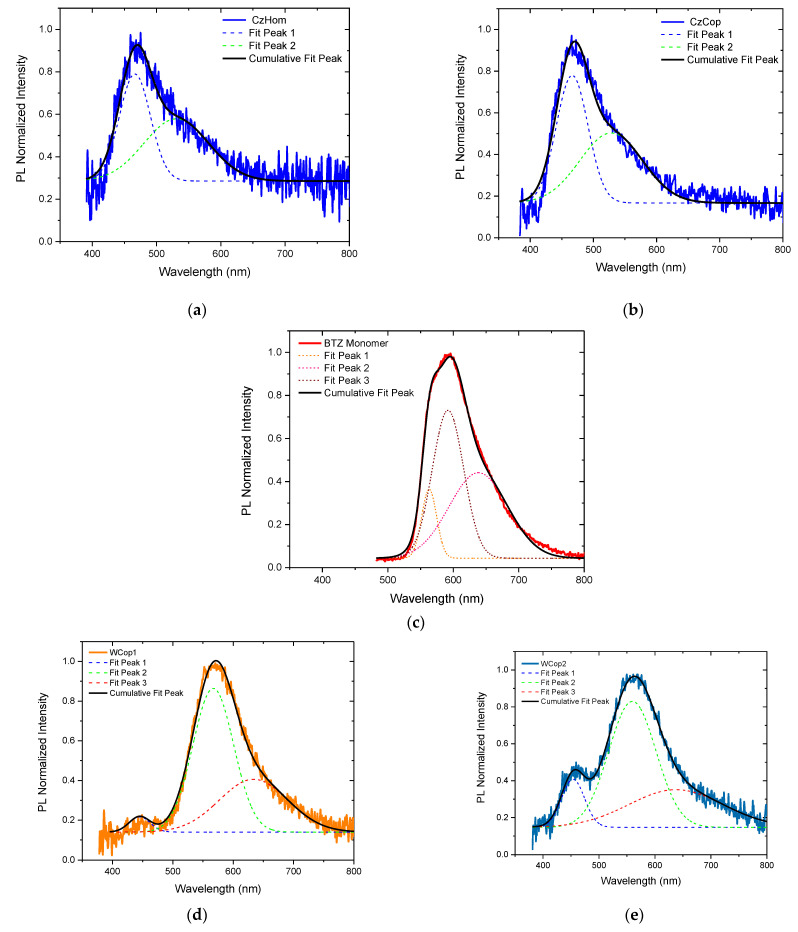
Deconvoluted normalized PL spectra of (**a**) CzHom, (**b**) CzCop, (**c**) BTZ monomer, (**d**) WCop1 and (**e**) WCop2 films.

The PL emission spectra of CzHom, CzCop, BTZ and WCop thin films, recorded upon excitation at 370 nm, are illustrated in Figure 6. For their better evaluation, a deconvolution fitting analysis of the experimental PL spectra was realized. Regarding the deconvolution analysis, two Gauss oscillators were used for CzHom and CzCop, whereas three Gauss oscillators were used for BTZ monomer and for WCop copolymers, respectively. Table 4 shows the results of this analysis, which are the wavelengths where the maximum of the PL emission of the films is located and the full width at half maximum (FWHM). The PL spectra of both CzHom and CzCop exhibit a dominant well-distinguished peak at λ^max^~466 nm (blue range) and a broader one at λ^max^~530 nm (green range). In the case of BTZ, it is obtained a dominant peak at λ^max^~592 nm (yellow range), a broader one at λ^max^~638 nm (red range) and a weak peak at λ^max^~563 nm (green range). Once the BTZ unit is incorporated into the Cz main chain, the characteristics of the PL emission alter. The PL emission profile of both white copolymer films is dominated by a broad band centered at 560–567 nm (green range), a less intense and broader one at 634–638 nm (red range), which can be ascribed to the contribution of BTZ chromophore excitation, as well as a third band ~445 nm (blue range) originated from the Cz units. It is observed the weak emission of the latter and its enhancement by increasing the Cz content in the WCop2 film.

It is notable that the broad emission of the copolymers WCop1 and WCop2 covers the visible range, which is generated from the individual emission of blue and red chromophores, and the energy transfer mechanism between them. In the case of WCop1 film, the emission of the blue chromophores was quenched, which is assigned to the efficient energy transfer from blue to red chromophore. More specifically, some photo-excited Cz units are deactivated partly by the energy transfer mechanism to BTZ units, resulting in the excitation of BTZ, which is followed by orange light emission from BTZ [11]. On the other hand, in WCop2, the emission of blue segments is significantly enhanced due to the incomplete energy transfer from blue emission units to red ones.

For WCop1 and WCop2 PL spectra, it is obvious that the orange emission is appeared due to the effective energy transfer mechanism from the Cz unit, as we discuss above. We can assume that both the polymer chain architecture and the percentage of dopant concentration influence the FET mechanism. According to H. Wang et al. [11], in a very lightly doped copolymer the inter-chain FET, is favored, whilst the intrachain FET is predominant for the highly doped copolymer. Based on this, it is proposed that the BTZ unit is excited by inter-chain FET from Cz, which is attributed to the promotion by π-stacking of the polymeric chains in aggregation. Thus, the close contact between chains favors interchain transport of the excited singlet species [11,17]. So, by controlling the ratios of the two chromophores, we achieve the emission of each chromophore and the partial energy transfer mechanism between them, leading to the desirable broad emission covering the whole visible range.

### 3.4. Electroluminescence

**Figure 7:**
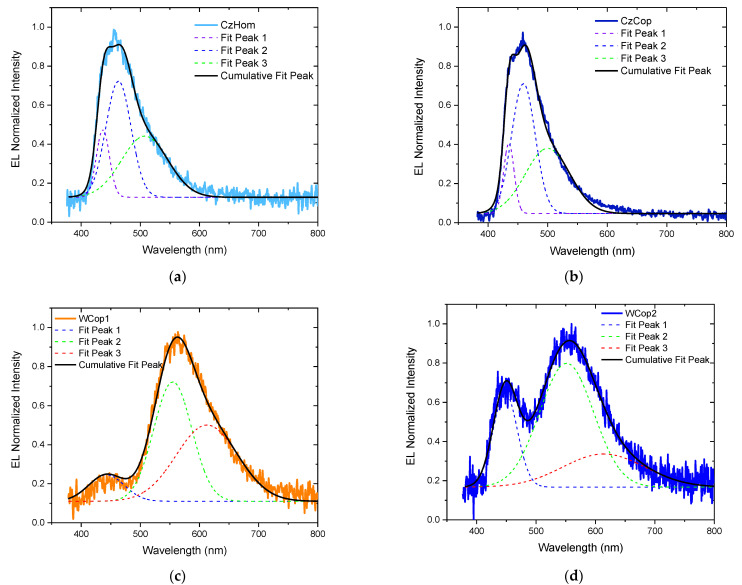
Deconvoluted normalized EL curves of (**a**) CzHom, (**b**) CzCop, (**c**) WCop1 and (**d**) WCop2 copolymers films.

**Figure 8:**
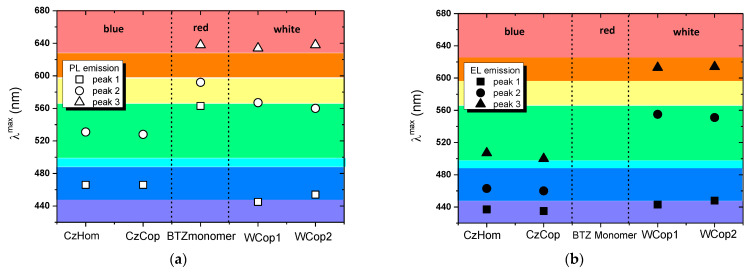
Schematical representation of the λ^max^ of the peaks calculated though the deconvolution analysis of the (**a**) PL spectra of the CzHom, CzCop, BTZ, WCop1 and WCop2 films, and (**b**) EL spectra of the CzHom, CzCop, WCop1 and WCop2 devices.

To investigate the Electroluminescence (EL) characteristics of the emitting polymers, we have fabricated OLED devices with the following architecture: Glass/ ITO/ PEDOT: PSS/ Emissive layer/ Ca/ Ag, as depicted schematically in Figure 1. The EL spectra were measured in the wavelength range from 380 to 800 nm by applying a bias voltage from 1 to 14 V with 1 V step. The respective EL spectra obtained at 12 V for the produced WCop devices are presented in Figure 7. For comparison, the EL spectra of CzHom and CzCop devices are also presented. The spectra were analyzed using the deconvolution procedure with an appropriate number of Gauss oscillators and the results concerning the wavelengths of the peaks λ^max^ and the broadenings FWHM are summarized in Table 4, whereas the λ^max^ are also presented graphically in Figure 8a (PL spectra analysis) and b (EL spectra analysis). As it can be seen, the produced devices of WCop exhibited broad emission covering the entire range of the visible spectrum. The EL emission of the WCop1 copolymer is found to originate from the BTZ group with the dominant peak at 554 nm and a significantly weaker emission peak at 442 nm attributed to Cz moiety. In the case of WCop2, it is also obtained the BTZ dominant peak at 551 nm, whilst the Cz peak at 448 nm is reinforced.

**Figure 9:**
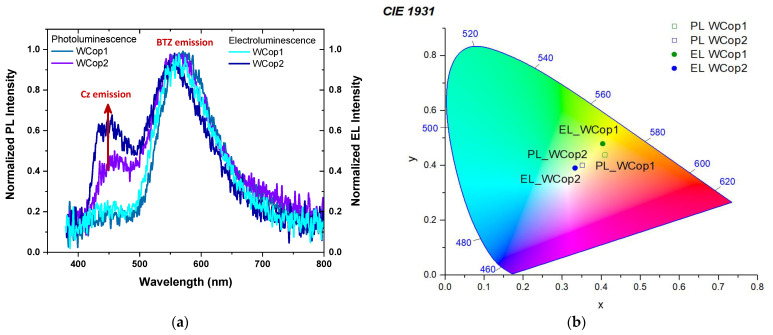
(**a**) Normalized PL and EL spectra of the studied white light emitting copolymer films. (**b**) CIE diagram of PL and EL emission of the studied white copolymers.

Similarly, to PL emission, EL emission of WCop1 is dominated by the characteristic emission of the BTZ units, as shown in Figure 9a. On the contrary, for the case of WCop2, two characteristic emission peaks exist in both PL and EL spectra, which are attributed to the Cz and BTZ segments, and it is notable to mention that the Cz emission is enhanced in EL spectra. Generally, the PL results from the direct photoexcitation of the emissive thin film and the recombination from the excited states, whereas the EL depends on the carrier injection mechanisms, the transportation, and the recombination of charges across the device structure. In this work, the PL measurements are conducted under the excitation source closer in the blue region. Thus, the efficiency of the photoexcitation of BTZ was expected to be low, so its emission occurs primarily because of the energy transfer from the Cz compound [9,13,31,32,33,34,35,36].

Yang et al. [37] proposed a strategy to achieve white light emission from a single-emissive layer in WOLED devices based on incomplete energy transfer between a blue-host and an orange emitting dye as a dopant. In their study, a deep blue-emitting beryllium complex bis (2-(2-hydroxyphenyl)-pyridine) beryllium (Bepp_2_) doped with a wide-bandwidth orange-emitting fluorescent dye 4-(dicyanomethylene)-2-methyl-6- (4-dimethylaminostyryl)-4-H-pyran (DCM) were used as the emissive layer. They observed that the competitive mechanisms between efficient energy transfer from the blue host to the orange dopant and exciton formation on the orange dopant by charge trapping would have a considerable effect on the relative emission intensity [37]. With an increase in blue-emission intensity, they achieve high-quality white light emission due to the incomplete energy transfer from Bepp_2_ to DCM. [37,38]. Hereby, one can see that in the EL emission of WCop2, the intensity of the Cz emission peak is higher than in PL spectra, as illustrated in Figure 9a. This may be owing to the double contribution of incomplete energy transfer between the two chromophores and more efficient direct charge-trapping of Cz [35,36]. The significant increase of the Cz concentration indicates again that both the energy transfer mechanism as well as the charge-trapping in the Cz unit leads to the balanced individual emission of each chromophore. This fact contributes to the achievement of a total emission towards the white light.

Additionally, the Commission Internationale de L’Eclairage (CIE) chromaticity coordinates of the EL spectral emissions from the WCop1 and WCop2 devices, presented in Figure 9b, obtain values at (0.40, 0.48) and (0.33, 0.38), respectively. The latter values are located at the white light region, and they are very close to the white point (0.33, 0.33). As a result, WCop2 is suggested as the most promising material for high purity white light. According to the CIE chromaticity coordinates of WCop1, both PL and EL emission exhibit red shift with increasing the BTZ unit in polymer chains in comparison to WCop2. H. Wang et al. [11] presented the investigation of the properties of copolymers, in which different concentrations of 4,7-bithienyl-2,1,3-benzothiadiazole (DBT) molecules were inserted, such as an orange light unit, in poly (9,9-dioctyl) fluorene (PFO) chain. They observed the same phenomenon in their study, in the case of copolymer with the higher concentration of orange unit, and they suggested that this fact is related to the interchain interactions [39].

**Figure 10:**
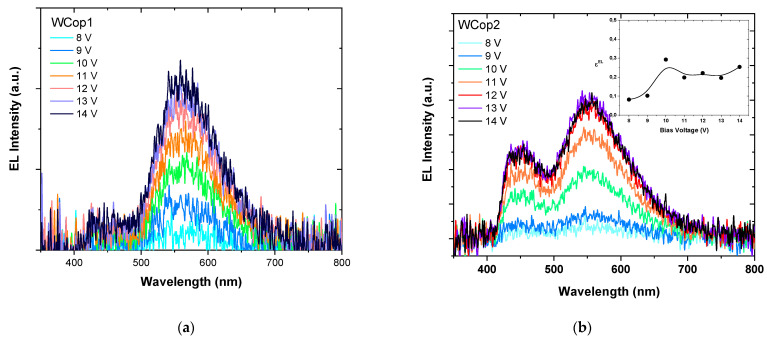
The evolution of EL emission spectra of copolymers (**a**) WCop1 and (**b**) WCop2 under excitation with various bias voltages. Inset in (**b**) shows the *ε*^EL^ parameter with the bias voltage.

We have further studied the effect of different applied voltages on the EL spectra of the fabricated WOLEDs. As illustrated in Figure 10a, EL spectra of WCop1 under different voltages contain a dominant emission band at around 560 nm (BTZ emission). The intensity of BTZ emission was found to increase by increasing the applied voltage, whereas the Cz emission remains negligible. This indicates that an effective energy transfer from the Cz segment to BTZ takes place leading to a broad featureless emission profile, having a maximum at the green-yellow color range, with increasing intensity with voltage [11]. Thus, it can be assumed that the electrons injected from the cathode are firstly trapped by the Cz segments and combine with holes to form excitons at low voltages. As the bias voltage is increased, the large number of excitons from the Cz unit deactivates by FET and excites BTZ, resulting in a dominant orange emission from the latter [11,40].

The evolution of EL profiles is different in the case of WCop2, which is presented in Figure 10b. As we can observe the Cz emission is present from the low bias voltages. However, the increase of bias leads to a well distinguished double-peak intensity profile. This fact leads us to speculate that the Cz unit in the copolymer plays the role of a trap site. Furthermore, saturation in the EL intensity is established for voltages above 12 V. To quantify the degree of energy transfer from Cz to BTZ units we calculated the relative intensity of the residual Cz emission appeared in EL spectra, *I*_CZ,_ by integrating the respective emission peak derived by the deconvolution analysis and dividing it by the total emission obtained by integrating the whole spectrum *I*_TOTAL_ [5,40]. The *ε^EL^* parameter accounts this ratio (*I*_CZ_/*I*_TOTAL_). The inset in Figure 10b depicts the results from this analysis. The low *ε^EL^* values at low bias voltages reveal a more effective energy transfer from Cz to BTZ. At higher voltages, it seems that the excitons are trapped by Cz units and afterwards a small amount of them transfer their energy to the BTZ units. This reveals an incomplete energy transfer between the chromophores leading to a sufficient broad emission covering the whole visible range [11,40].

It is remarkable that considering the peak intensities in EL spectra, blue emission is strong enough to render color balanced between blue and red-orange-green emission to achieve white light. This phenomenon is important to obtain WOLEDs with a wide wavelength EL spectrum. Thus, by managing the incomplete energy transfer from host to guest in the emissive layer, the host material can also act as the blue emitter and produce blue emission, when the concentration of guest is generally low (e.g., <1%) to ensure the white light emission [38].

### 3.5. Electrical Characteristics of OLED Devices

**Figure 11:**
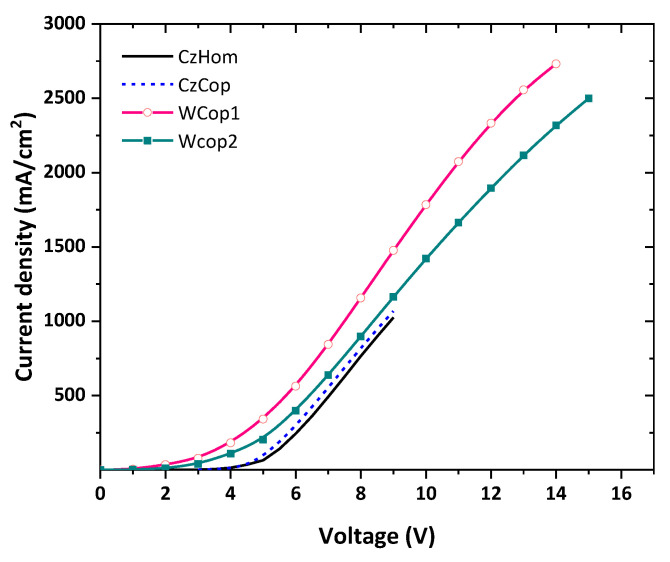
Current density-voltage characteristic curves of CzHom, CzCop and WCop1, WCop2 devices.

Generally, the injection and transport mechanisms of current carriers in the device are quite complicated phenomena as they are influenced by numerous parameters, such as charge carrier mobility, a Schottky injection barrier, thickness, dielectric constant, energy levels, density of charge traps and applied voltage. The electrical characteristics of the fabricated OLED devices were evaluated by measuring the current density *J* versus the applied bias voltage *V* and the results are depicted in Figure 11. It can be easily derived that the white emitting copolymer devices exhibit higher current density compared to Cz homopolymer and copolymer devices. It is also observed that the current density threshold voltages of the white copolymer WCop1 and WCop2 devices shift to lower values, from 4 to 2 V. This could be ascribed that the electron injection becomes more efficient, as the LUMO level of the emissive layer decreases and is closer to the work function of Ca (about 2.8 eV). As a result, a more balanced injection and transport of both types of carriers takes place and a decrease in the turn-on voltage of the OLEDs is obtained [12,41]. Moreover, it is important to mention that a small increase of current density is observed for CzCop, in comparison to CzHom. This confirms that the 2,6-bisphenyl-pyridine (Py) moiety, which has been incorporated into the Cz chain, improves the carrier mobility in the organic semiconductors [42].

**Figure 12:**
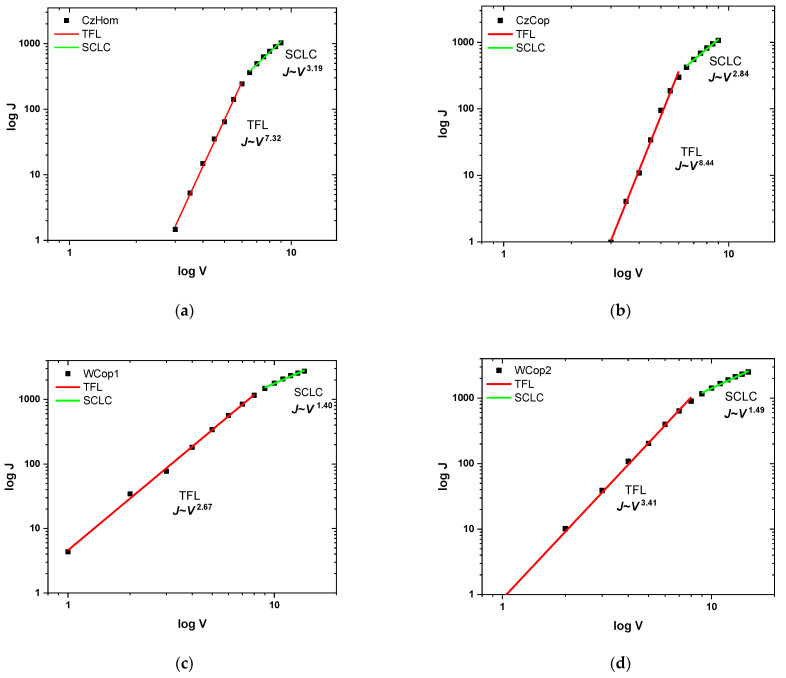
Logarithmic plots of current density-voltage characteristic curves of (**a**) CzHom, (**b**) CzCop, (**c**) WCop1, and (**d**) WCop2 devices with the respective analysis.

The *J*-*V* curves in a logarithmic scale for CzHom and CzCop as well as of WCop1 and WCop2 are presented in Figure 12a–d, respectively. Further analysis of the *J*-*V* characteristics enables the evaluation of the carrier mobility in the respective devices. In general, *J* and *V* relationship in a semiconducting layer is expressed by $J\propto V^{m}$ and has been known to be composed of several distinctive regions, which are defined by the value of exponent *m*. More specifically, these regions include: (a) the low-voltage zone, that corresponds to ohmic conduction across the sandwich structure Ohmic current, where *J* is linearly proportional to *V* (J∝V), (b) the Trap-limited Space Charge Limited Current (T-SCLC) zone, where the exponent is around 1–2 ($J\propto V^{1–2})$, (c) the Trap-Filled Limited (TFL) current zone, being characteristic of amorphous materials, in which induced traps are filled with charges and the exponent is between 2 and 100 ($J\propto V^{2–100}$) and finally (d) the Space Charge Limited Current (SCLC) zone, which is the high-voltage zone where the exponent is close to 2 ($J\propto V^{2}$) and it is dominated by the trap filling process since the increase of current causes a gradual filling of the states [43,44,45,46,47,48,49,50].

In our results, we cannot distinguish the Ohmic and T-SCLC regions. The Ohmic region does not appear due to the high barrier height between the polymer and metal interfaces. For example, the Ohmic region appears if conducting polymer is sandwiched between two electrodes and both electrodes offer low barrier height (~ohmic response) to the polymer-metal interface, then the injected carrier from the electrode forms a space charge region consisting of a large number of injected carriers and equilibrium free carriers inside the polymer. The T-SCLC region also does not appear, as the trap energy level becomes deeper even with traps of a single energy level, a fact that leads to the formation of the distinctive TFL region. As the trap density is increased the T-SCLC current density starts to decrease and the distinctive TFL region starts to form.

For the carbazole homopolymer CzHom and copolymer CzCop devices, the *J*-*V* characteristic curves exhibit higher slopes in the TFL and SCLC in comparison to those of the white light emitting copolymers. The TFL zone is dominated by the trap filling process and the increase of current causes a gradual filling of the states and subsequently a rise of the activation energy. Especially, in the case of CzHom and CzCop devices, the slope of TFL zone (3–6 V) is ~7.32 and ~8.44, respectively, whereas in the case of the WCop1 and Wcop2 devices (1–8 V) it is ~2.87 and ~3.41, respectively. The difference in the voltage range of the TFL zone between the devices is related to their different turn-on voltage (4 V for CzHom and CzCop to 2 V for Wcop1 and Wcop2) due to the higher activation energy [43,44,45].

It is established that polymers have a unique charge transport mechanism as a combination of delocalization and localization of charge carriers with intramolecular and intermolecular charge interaction, respectively. Localization of charge carriers can be caused by other sources of disorder such as chemical impurities and structural defects. In addition, these sources can lead to the formation of electronic states in the bandgap of the Organic Semiconductor (OSC). These in-gap states can subsequently trap charge carriers and hinder their transport, further preventing the OSC from realizing their intrinsic mobilities. The nature of traps distribution inside the polymer is varied and depends on many factors such as the nature of the material itself, the polymerization process, the nature of dopants, and the solvent. Generally, traps are classified as (i) shallow traps and (ii) deep traps for both electron and hole. If traps are very close to the conduction band (LUMO) for electrons or in the vicinity of the valence band (HOMO) within the energy bandgap, then traps are classified as shallow traps for electrons and holes, respectively. On the other hand, deep traps of electrons and holes exist far away (mid of the energy bandgap) from the conduction (LUMO) and valence (HOMO) bands, respectively [46,49]. As noted above, the region TFL is mainly determined by the density of charge traps. Thus, it is obvious that the slope of the TFL region is stiffer for CzHom and CzCop. These results may imply that the energy level of traps is deeper in comparison to Wcop1 and Wcop2. On the other hand, in the case of the white emitting copolymers, the exponent of the *J*-*V* curve in the TFL region is lower and this could be ascribed to the shallow energy level of the charge traps [43,47,50].

In the high-voltage zone SCLC, where the exponent is around 2, the space-charge-limited current (SCLC) is reported, and it is ascribed to free charges after complete trap filling. When transport in a trap-free emissive polymer is not injection limited, i.e., the current is limited due to space-charge accumulation, the mobility of the charge carriers can be extracted from current versus voltage curves, for the emissive layer sandwiched between two conducting electrodes.

As we have already mentioned above, the transport of carriers in the emissive layer within this region is controlled by the trapping and detrapping of carriers at both energetic and positional distribution. Traps are impurities and/or structural defects which provide localized states between HOMO and LUMO of the emissive layer. These localized states trap the free carriers and prevent them from taking any role in the charge transport process leading to the degradation of the electrical properties of the polymer as well as the device’s performance. When the applied voltage is higher than the average energy associated with traps density, then a trap-free space charge-limited current is created [47]. Therefore, the density of localized states is one of the main factors affecting carrier mobility.

The doping efficiency is governed by several factors such as the offset of energy levels between the host and the guest and the dopant concentration. The mechanism of charge transport in doped OSCs is complex and is dominated by several competing processes that depend on the aforementioned factors. For example, the addition of a dopant can either broaden the density of states of the host thereby introducing tail states, or the dopant-induced charge carries can fill up existent trap states to neutralize them or the presence of the dopant can annihilate the trap states. In this study, the host–guest of the Cz-BTZ in a copolymer would imply that charge mobility is favored [47,50].

**Figure 13:**
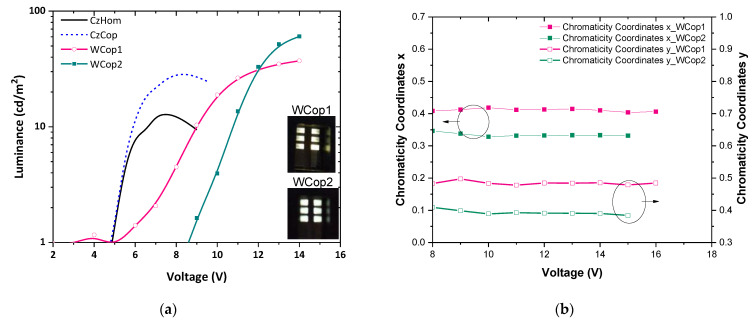
(**a**) Logarithmic plot of Luminance-voltage characteristics. The inset presents the photographs of WCop1 and WCop2 devices operating at 14V. (**b**) The dependence of the CIEx and CIEy coordinates on the Voltage of the fabricated OLEDs.

Figure 13a presents the evolution of the luminance–voltage of the fabricated OLEDs and their electrical characteristics are shown in Table 5. The maximum luminance measured for CzHom is 12 cd/m^2^ at 8 V, and for CzCop is 28 cd/m^2^ at 8 V as well. In addition, the maximum luminance measured for Wcop1 and Wcop2 is 40 cd/m^2^ and 60 cd/m^2^ at 14 V, respectively. The inset presents the photos of Wcop1 and Wcop2 devices operated at 14 V, from which the emission homogeneity is demonstrated.

While the applied voltage is increasing, the color point of white light emission remains stable, as is shown in Figure 13b. The CIE coordinates of Wcop1 changed only slightly from (0.408, 0.483) at 8 V to (0.404, 0.479) at 15 V and in the case of Wcop2 changed from (0.346, 0.409) at 8 V to (0.332, 0.384) at 15 V. These results verify that the values of CIE chromaticity coordinates are constant and independent of increased voltage. Specifically, the copolymers based on Cz unit as polymeric hosts with blue emission benefit from the other blue emitting polymers, such as Polyflourene. Among the main advantages are the wide bandgap of Cz and its outstanding thermal, photochemical, and chemical stability [10]. These properties make Cz an attractive candidate to design hosts for single emission white polymers. Whereas Polyflourene units suffer from poor color purity and stability during the device operation and this fact is discussed in the group of Wang et al. [11]. One of the main challenges for the white OLEDs is to obtain voltage-independent EL spectra, avoiding undesirable shifts in the white emission CIE coordinates for different values of driving voltages. Furthermore, the Color Rendering Index (CRI) for the Wcop2 device is 70, which is promisingly high for solution-processed white OLEDs while for the Wcop1 device is 60. In the same way, the correlated color temperature (CCT) is 4063 K and 5498 K for Wcop2 and Wcop1 devices, respectively. Thus, the Wcop1 device emits neutral white light and the Wcop2 device emits cool white light [36,37]. These properties effectively contribute to the achievement of a total emission towards the desirable white light and for this reason, these novel copolymers are promising candidates for white light applications.

## 4. Conclusions

To summarize, we have developed synthetically versatile semiconducting polyether sulfones that can modulate different chromophore combinations, are of high molecular weights, are easily scalable and highly soluble and afford stable inks in common organic solvents like DMF. We have demonstrated the fabrication of solution-processed WOLEDs (ITO/ PEDOT: PSS/Copolymers/Ca/Ag) with the single-emissive layer structure based on novel copolymers bearing blue (carbazole-Cz) and red (benzothiadiazole-BTZ) chromophores. We have studied the optical and photophysical properties of two copolymers having different ratios and for comparison the carbazole homopolymer, the carbazole copolymer and the benzothiadiazole monomer. It was achieved broadband EL emission covering the visible range, with promising CRI values and stable color point in the white region under different applied voltages. We have also examined the current-voltage response of the devices, which allows us to estimate the carrier mobility of the novel copolymers. Preliminary device characteristics are promising, however, there is a need for further investigation to improve the functionalization of the produced WOLED devices towards the achievement of higher efficiency.

## Figures and Tables

**Table 1:** Molar ratios of Copolymers that were synthesized in this work.

Polymer	Co-Monomers Ratio ^a^		
	Py (*x*)	Cz (*y*)	BTZ (*z*)
CzHom	0	1	0
CzCop	0.5	0.5	0
WCop1	0.5	0.45	0.05
Wcop2	0.5	0.495	0.005

^a^ Based on the monomers’ feed ratio.

**Table 2:** Data obtained from Gel Permeation Chromatography (GPC) ^a^ of the synthesized polymers.

Polymer	Mp ^b^	Mn ^c^	PD ^d^
CzCop	59,300	34,700	1.50
WCop1	74,900	49,800	1.77
WCop2	53,100	34,900	1.62

^a^ Determined by GPC with a UV detector, CHCl_3_ as eluent, versus polystyrene standards. ^b^ Mp = peak molecular weight at the peak of the distribution curve. ^c^ Mn = number-average molecular weight. ^d^ Disp = polydispersity Mw/Mn.

**Table 3:** The calculated best-fit results derived by the analysis of the measured $\langle \tilde{\varepsilon }\left(E\right)\rangle$ spectra of the films.

	Thickness (nm)	Optical Band Gap (eV)		Electronic Transition Energy (eV)					
		$E_{g}^{TL}$	$E_{g}^{Tauc}$	*E*_01_	*E*_02_	*E*_03_	*E*_04_	*E*_05_	*E*_06_
CzHom	23	2.78	3.00	3.05	4.02	4.57	5.28	6.20	9.12
CzCop	27	2.67	2.95	3.08	4.02	4.53	5.29	6.18	9.29
WCop1	11	2.57	2.93	3.10	3.96	4.50	5.62	6.13	9.35
WCop2	32	2.73	2.96	3.09	3.99	4.57	5.39	6.23	9.16

**Table 4:** Results of the deconvolution analysis of the PL and EL spectra.

		PEAK 1		PEAK 2		PEAK 3	
	Spectrum	λ^max^ (nm)	FWHM (nm)	λ^max^ (nm)	FWHM (nm)	λ^max^ (nm)	FWHM (nm)
CzHom	PL	466	57	531	119		-
	EL	437	22	463	42	507	79
CzCop	PL	466	59	528	117	-	-
	EL	435	18	460	40	500	75
BTZ monomer	PL	563	27	592	56	638	105
WCop1	PL	445	35	567	68	634	113
	EL	443	60	555	64	613	104
WCop2	PL	454	41	560	84	638	168
	EL	448	41	551	91	614	138

**Table 5:** The electrical–operational characteristics of fabricated OLED devices.

	Current Density Turn-on Voltage (V)	Luminance Turn-on Voltage (V)	Luminance (cd/m^2^)	CRI	CCT (K)
CzHom	4	5	12		
CzCop	4	5	28		
Wcop1	2	5	40	60	4063
Wcop2	2	8.8	60	70	5498

## Data Availability

Data presented in this article is available on request from the corresponding author.
